# M2 Macrophage Co-Expression Factors Correlate With Immune Phenotype and Predict Prognosis of Bladder Cancer

**DOI:** 10.3389/fonc.2021.609334

**Published:** 2021-03-22

**Authors:** Yutao Wang, Kexin Yan, Jianfeng Wang, Jiaxing Lin, Jianbin Bi

**Affiliations:** ^1^ Department of Urology, China Medical University, The First Hospital of China Medical University, Shenyang, China; ^2^ Department of Dermatology, China Medical University, The First Hospital of China Medical University, Shenyang, China

**Keywords:** M2 macrophage, weighted gene co-expression network analysis, tumor-associated macrophages, immune phenotype, tumor mutation burden

## Abstract

**Purpose:**

Therapeutic targets of tumor-associated macrophages have been discovered and used clinically as immunotherapy. M2 macrophages are tumor-associated macrophages that promote cancer progression. This article explores the related factors and the effects of type M2 macrophages.

**Method:**

We obtained bladder cancer (BC) sequencing data from TCGA and GSE31189. We used the CIBERSORT algorithm calculate M2 macrophage proportions among 22 type immune cells. The *Estimate* package was used to measure BC purity. M2 macrophage-related genes were selected using WGCNA. Receiver operating characteristic curves and Kaplan–Meier analyses were performed to determine the risk score, conducted for M2 macrophage-related factors. The Pearson test was used to determine the correlation among M2 macrophage-related genes, clinical phenotype, immune phenotype and tumor mutation burden (TMB). The TIMER database was used to calculate correlations among M2 macrophages and other cancers.

**Results:**

Expression of four M2 macrophages co-expressed genes (CD163, CD209, CSF1, MMD) positively correlated with infiltration of M2 macrophages, which were enriched in the negative regulation of immune system process and the positive regulation of tumor necrosis factor production. M2 macrophage-related factors are robust biomarkers for predicting the BC and immune phenotypes. The Cox regression model built on these four co-expression factors showed a close correlation with outcome (AUC = 0.64). The four co-expression factors negatively correlated outcome and TMB.

**Conclusion:**

Four co-expressed genes promote high levels of infiltration of type M2 macrophages in the negative regulation of immune system processes and the positive regulation of tumor necrosis factor production processes. These co-expressed genes and the biological process they involve might suggest new strategies for regulation of chemotaxis in M2 macrophages.

## Introduction

Bladder cancer (BC) ranks fourth among the most common tumors in developed countries (1). In the past 15 years, tumor-related mortality of breast cancer, prostate cancer, colorectal cancer, and lung cancer have decreased by 20–40%, while the mortality of BC has decreased by less than 5% (2). Despite adopting various strategies to treat BC, the outcome of advanced BC has not significantly improved (2). Therefore, immunotherapy for advanced BC has attracted increasing attention.

BC is characterized by a high mutation rate and many new tumor antigens, and it is relatively sensitive to immunotherapy (3). Immune regulation plays a key role in BC. This process includes immune checkpoints (primarily programmed cell death 1 [PD-1 and programmed cell death 1 ligand 1 [PD-L1), as well as regulatory T cells, original source of suppressor cells and tumor-associated macrophages (TAM; macrophages in primary or secondary tumor tissues) and type 2 innate and adaptive lymphocytes (4). Clinical and experimental evidence suggested that macrophages promote the progression and metastasis of solid tumors (5, 6). Targeting macrophages has become a new treatment strategy (7, 8). TAMs are divided into two types, M1 and M2 (9).

M1 macrophages, namely classically activated macrophages, highly express IL12, IL23, nitric oxide, reactive oxygen molecules, dissolve tumor cells, present tumor antigens to T cells, produce immunostimulatory factors, and promote CD8 T cell activation. Enhance their anti-tumor effects (10). However, M2 type macrophages, namely alternatively-activated macrophages, promote tumor formation and development. In addition, M2 type macrophages cannot present tumor antigens (10). Therefore, compared to M1 type macrophages, T cell function is relatively inhibited. Nevertheless, the mechanism of this polarization of macrophages is not clear. This article explored the immune-related factors of M2 macrophages in BC and constructed a co-expression network of M2 macrophages using the WGCNA method.

## Method

### Data Acquisition and Processing

We downloaded The Cancer Genome Atlas TCGA-BLCA FPKM data (http://cancergenome.nih.gov/) containing 414 cancer tissue samples and 19 normal tissues. GSE31189 (11) was also downloaded from the GEO (http://www.ncbi.nlm.nih.gov/geo/) database whose platform is GPL570. GSE31189 contained 52 urothelial cancer samples and 40 non-cancer samples. Genomic, transcriptomic, and clinical information from patients with metastatic urothelial cancer treated with an anti-PD-L1 agent (atezolizumab) were obtained under the Creative Commons 3.0 license and can be downloaded from http://research-pub.gene.com/IMvigor210CoreBiologies (12). In subsequent studies, we used cancer samples from the cohort for analysis. We calculated M2 macrophage cell proportions based on the LM22 matrix and CIBERSORT (13) algorithm. Bladder samples with P < 0.05 were considered to be significant and were determined in the subsequent analysis.

### M2 Macrophage Co-Expression Network Construction

WGCNA is a systems biology approach that converts co-expression correlations into connection weights or topology overlap values (14). We used it to selected M2 macrophage cell co-expressed genes. The expression patterns were similar for genes involved in the same pathways or biological processes (15). To build a scale-free topology network, we set the soft threshold at 5 both in TCGA and GSE31189, R square = 0.94 in TCGA, R square = 0.88, and the number of genes in the minimum module at 30. We input the M2 macrophage cell proportions as phenotype files. In this manner, a cluster of M2 macrophage cell proportion-related genes with similar functions were identified using WGCNA (16). Subsequently, the same analysis process was carried out in GSE31189 and the factors with M2 correlation greater than 0.4 in the most relevant modules were intersected.

### Intersection Function Analysis

The genes were selected using Pearson correlation coefficient > 0.4 between genes and M2 macrophage cell proportions. The Database for Annotation, Visualization and Integrated Discovery (DAVID, v6.8) is a function-enrichment tool that supplies biological explanations of gene lists and proteomic studies obtained from high-throughput sequencing (17). We used the Kyoto Encyclopedia of Genes and Genomes (KEGG) (https://www.genome.jp/kegg/) (18) and Gene Ontology (GO) (http://geneontology.org/) analysis (19) to identify the biological function in each co-expression module, such as biological processes, cellular components and molecular functions.

### Prognostic Value of Infiltration-Related Genes

We performed survival analysis on these intersection factors, and the factors with significant survival analysis results were included in the risk model. We verified the differential expression of M2 macrophage-related factors in various clinical stages and survival statuses. TMB is a measure of the total number of mutations per megabyte in a chromosome. This includes the total number of base substitution inserts, gene coding errors and deletions. The detail information could be obtained from our previously published articles (20). In addition, we calculated the TMB and tumor purity in TCGA samples, and explored the correlations between macrophage-related factors, TMB, and tumor purity (21).

### Pan-Cancer Analysis in TIMER

The Tumor Immune Estimation Resource (TIMER; https://cistrome.shinyapps.io/timer/) is a web resource for systematic evaluations of clinical impacts of various immune cells in diverse cancer types (22). It was used to analyze the correlations between CD8+ T cells and 33 types of cancer. Pearson correlation coefficient > 0.4 was considered significant.

### Gene Set Enrichment Analysis

Gene set enrichment analysis (GSEA) is a calculation method that determines the significance and consistency differences of a predefined dataset between two biological states (23). The gene matrix in TCGA was divided into high and low expression groups, in accordance with the median expression level of M2 macrophage infiltration-related genes. Based on allocation, biological functions related to the high expression group were identified, allowing us to identify the mechanisms underlying the role of M2 macrophage infiltration-related co-expression genes.

## Results

### M2 Macrophage Evaluation

**Figure 1:**
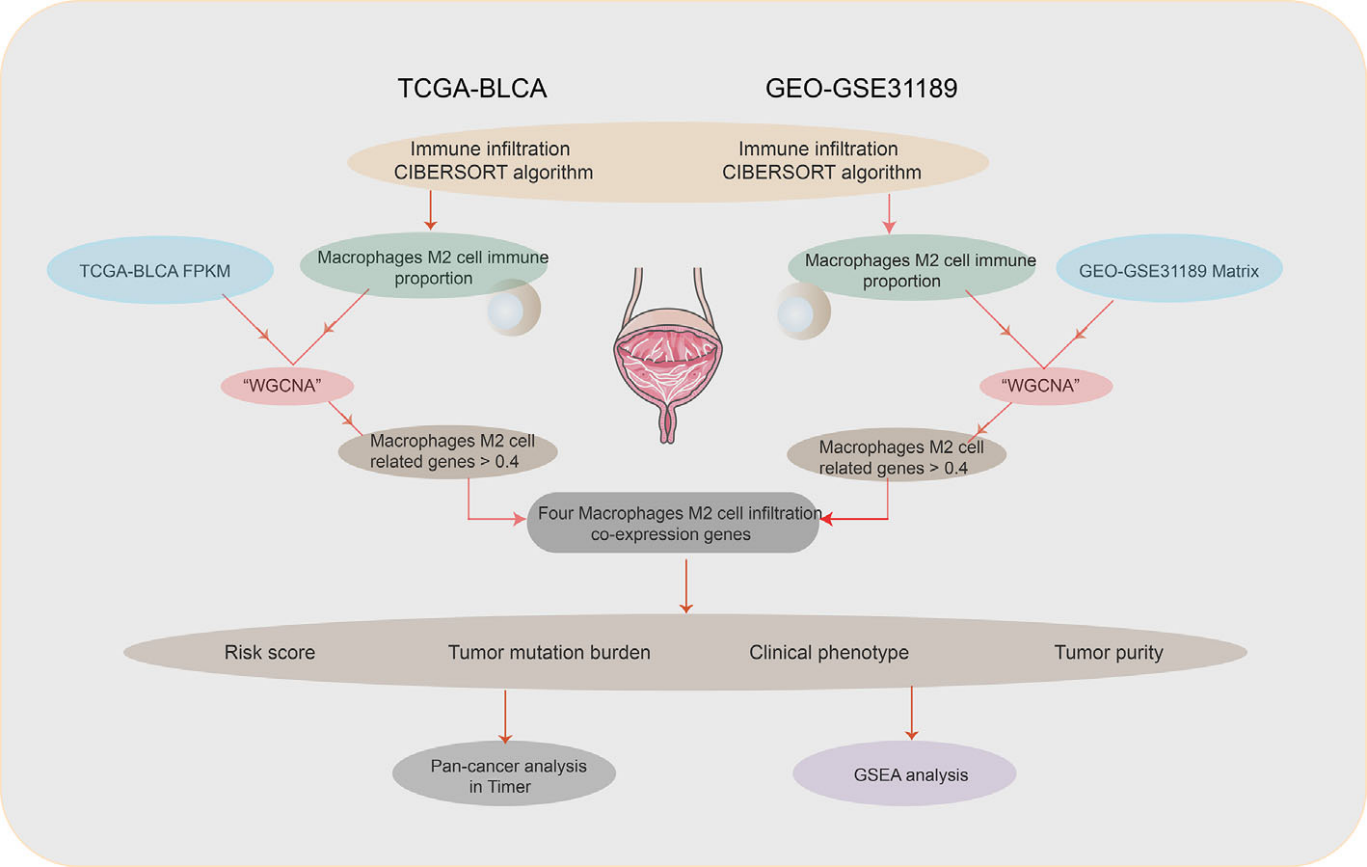
Flowchart.

**Figure 2:**
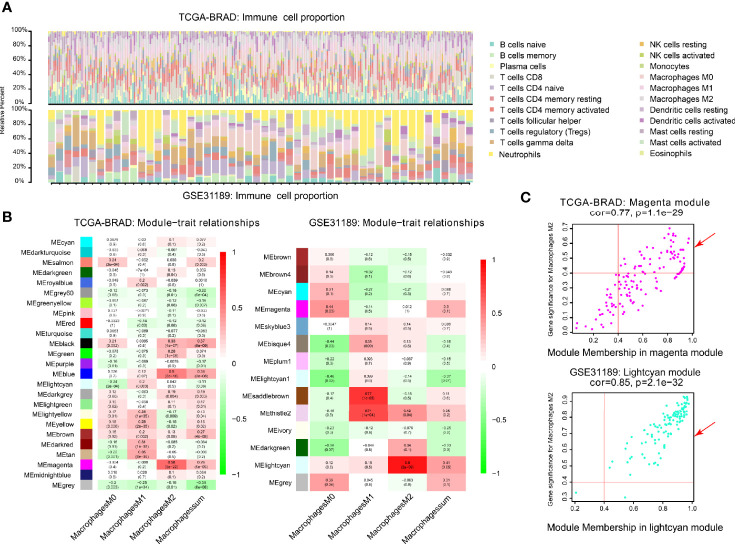
Co-expression network generated using WGCNA. **(A)** Immune proportion of TCGA-BLCA; Immune proportion of GEO-GSE31189. **(B)** Heatmap of module and immune cell proportions of TCGA and GSE31189. **(C)** The correlations between TCGA magenta module membership and M2 macrophage-related gene significance; The correlation between GSE31189 lightcyan module membership and M2 macrophage-related gene significance.

The flowchart of this paper is found in **Figure 1**. The CIBERSORT method was applied to assess the immune cell proportion. P < 0.05 was considered to be significant. Immune cell proportions of TCGA-BLCA and GSE31189 are found in **Figure 2A**. The results showed that the macrophage proportions were the highest in bladder tumor microenvironment. To determine the type of immune cell that is the most prognostic, univariate Cox regression analysis was performed. M2 macrophages acted as risk prognosis factor (**Table 1**). In this paper, we focused on factors co-expressed with M2 macrophages. Then, we summed the M2 macrophages into clinical phenotype data, and input these as phenotype profile in the subsequent analysis.

**Table 1:** Univariable Cox regression of immune cell proportions in bladder cancer.

Genes symbol	HR	HR.95L	HR.95H	pvalue
Macrophages M2	2.97E+01	2.89E+00	3.06E+02	4.33E-03
T cells CD8	2.69E-02	1.95E-03	3.71E-01	6.89E-03
Neutrophils	9.74E+04	1.61E+01	5.87E+08	9.70E-03
Macrophages M0	5.52E+00	1.15E+00	2.65E+01	3.26E-02
T cells follicular helper	7.71E-04	8.00E-07	7.43E-01	4.09E-02
T cells CD4 memory activated	2.44E-02	4.31E-04	1.38E+00	7.15E-02
Mast cells resting	3.81E+01	3.36E-01	4.32E+03	1.32E-01
Monocytes	1.24E+04	4.80E-02	3.21E+09	1.38E-01
B cells naive	1.49E-01	1.08E-02	2.06E+00	1.56E-01
Mast cells activated	3.73E+01	4.42E-02	3.15E+04	2.92E-01
Macrophages M1	1.36E-01	2.53E-03	7.31E+00	3.27E-01
T cells gamma delta	1.44E-05	1.01E-15	2.06E+05	3.50E-01
Plasma cells	1.32E-01	1.52E-03	1.16E+01	3.75E-01
Dendritic cells activated	4.14E+00	1.36E-01	1.26E+02	4.15E-01
NK cells activated	5.33E-02	1.24E-05	2.28E+02	4.92E-01
T cells CD4 memory resting	2.63E+00	1.41E-01	4.92E+01	5.17E-01
Eosinophils	1.35E+04	3.14E-10	5.79E+17	5.53E-01
T cells regulatory (Tregs)	2.51E-01	1.12E-04	5.64E+02	7.26E-01
Dendritic cells resting	6.47E-01	1.44E-02	2.91E+01	8.22E-01
T cells CD4 naive	1.66E-05	1.84E-47	1.50E+37	8.23E-01
B cells memory	1.48E+00	1.40E-02	1.56E+02	8.69E-01
NK cells resting	6.25E-01	5.89E-04	6.63E+02	8.95E-01

HR, hazard rate.

### Co-Expression Network of M2 Macrophage

We performed WGCNA analysis in TCGA-BLCA and GSE31189. The correlation coefficients between each phenotype and co-expression module of TCGA and GSE31189 are shown in **Figure 2B**. The magenta module had the strongest correlation with M2 macrophage proportions in TCGA-BLCA (Cor = 0.58; P= 6e-22) (**Figure 2B**). The lightcyan module had the strongest correlation with M2 macrophage proportions in the GSE31189 cohort (Cor = 0.9; P= 2.e-9) (**Figure 2B**). Based on these findings, we supplemented the heat map of the correlation between the factors in the magenta module and lightcyan module (**Figure 2C**; the module correlation coefficient was greater than 0.4; the M2 macrophage correlation coefficient was greater than 0.4)

### Intersection Function Analysis

**Figure 3:**
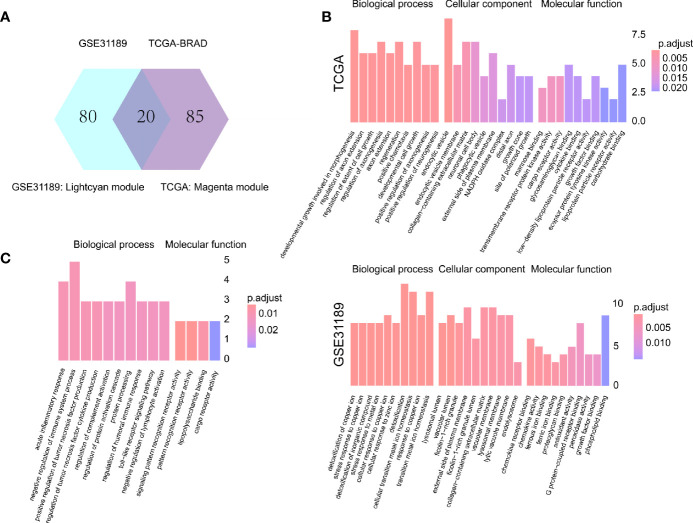
Intersection and functional analysis. **(A)** Venn diagram. **(B)** Functional enrichment of TCGA magenta module genes and GSE31189 lightcyan genes. **(C)** Functional enrichment of intersecting genes.

One hundred M2 macrophage co-expressed genes were identified with coefficient > 0.4 in the GSE31189 lightcyan module. We further identified 105 M2 macrophage co-expressed genes with coefficients > 0.4 in TCGA - BLCA lightcyan module. Then, 20 co-expression genes were determined by the intersection part of these two modules (**Figure 3A**).The M2 macrophage proportion correlation (TCGA) for these 20 factors are shown in **Table 2**. The M2 macrophage proportion correlations (GSE31189) for these 20 factors are shown in **Table 3**. The genes in TCGA - BLCA magenta module were significantly enriched in developmental growth involved in morphogenesis and cytokine binding (**Figure 3B**). The genes in GSE31189 lightcyan module were most significantly enriched in chemokine receptor binding (**Figure 3B**). The genes in the intersection part were most significantly enriched in the negative regulation of immune system process, regulation of tumor necrosis factor production and regulation of complement activation (**Figure 3C**).

**Table 2:** The correlation of intersecting genes with M2 macrophages in GSE31189.

ID	GS.Macrophage.M2	P - value
CD163	0.92	1.57E-10
VSIG4	0.92	5.83E-11
CSF1	0.85	<0.0001
MS4A6A	0.83	<0.0001
MPEG1	0.76	<0.0001
FGL2	0.51	0.009
CD14	0.66	<0.0001
TNS3	0.78	<0.0001
RNASE1	0.48	0.01
CD209	0.79	<0.0001
C3AR1	0.69	<0.0001
MS4A7	0.76	<0.0001
C1QB	0.89	8.99E-09
DPYSL2	0.85	<0.0001
LGMN	0.88	2.04E-08
MMD	0.47	0.02
KCTD12	0.93	5.85E-11
TYROBP	0.72	<0.0001
TPP1	0.75	<0.0001
LY96	0.53	0.007

GS, Gene significance.

**Table 3:** The correlation of intersecting genes with M2 macrophages in TCGA-BLCA.

ID	GS.Macrophage.M2	P - value
CD163	0.67	5.6E-31
VSIG4	0.65	1.43E-29
CSF1	0.65	1.73E-29
MS4A6A	0.63	5.44E-27
MPEG1	0.61	7.57E-25
FGL2	0.57	3.06E-21
CD14	0.56	3.1E-20
TNS3	0.56	3.66E-20
RNASE1	0.55	2.27E-19
CD209	0.54	4.31E-19
C3AR1	0.53	5.32E-18
MS4A7	0.52	1.63E-17
C1QB	0.50	2.09E-16
DPYSL2	0.50	5.02E-16
LGMN	0.48	1.23E-14
MMD	0.45	6E-13
KCTD12	0.45	9.47E-13
TYROBP	0.43	1.03E-11
TPP1	0.42	1.96E-11
LY96	0.40	2.34E-10

GS, Gene significance.

### Survival and Prognostic Analysis

**Figure 4:**
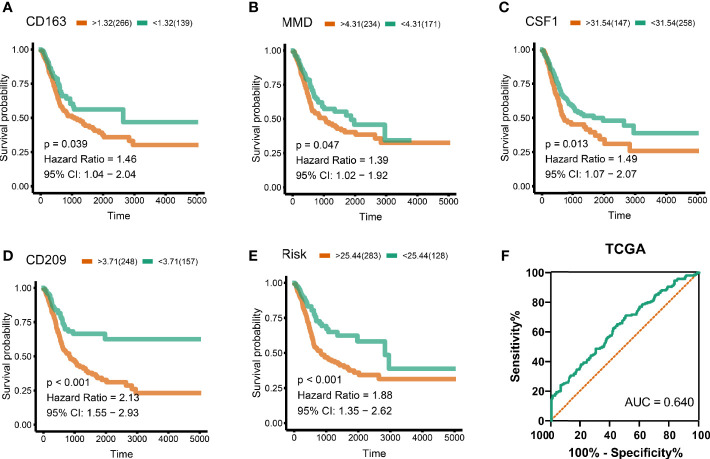
Survival analysis and risk score. **(A)** CD163 (TCGA: P = 0.039), **(B)** MMD (TCGA: P = 0.047), **(C)** CSF1 (TCGA: P = 0.013), **(D)** CD209 (TCGA: P < 0.001). Cox regression risk score model based on these genes. Risk score = 1 * CD163 + 1 * CD209 + 1 * CSF1 + 1 * MMD. **(E)** The patients in high risk groups for bladder patients (TCGA: P < 0.001; HR = 1.88) showed survival risk against low expression groups. **(F)** Area under curve = 0.640.

To analyze their influence on overall survival, we performed survival analysis. The patients in high-expression groups for CD163 (TCGA: P = 0.039) (**Figure 4A**), MMD (TCGA: P = 0.047) (**Figure 4B**), CSF1 (TCGA: P = 0.013) (**Figure 4C**), and CD209 (TCGA: P < 0.001) (**Figure 4D**) showed survival advantages against low-expression groups. Then, we generated a multi-Cox regression risk score model based on these genes: Risk score = 1 * CD163 + 1.007 * CD209 + 1.028 * CSF1 + 1.012 * MMD. The patients in higher risk groups for BC (TCGA: P < 0.001; HR = 1.88) (**Figure 4E**) showed survival risk against low expression groups, with AUC = 0.640 (**Figure 4F**). These results indirectly identified M2 macrophages related genes and M2 macrophages acted as crucial prognosis genes in BC.

### Clinical Phenotype Correlation

**Figure 5:**
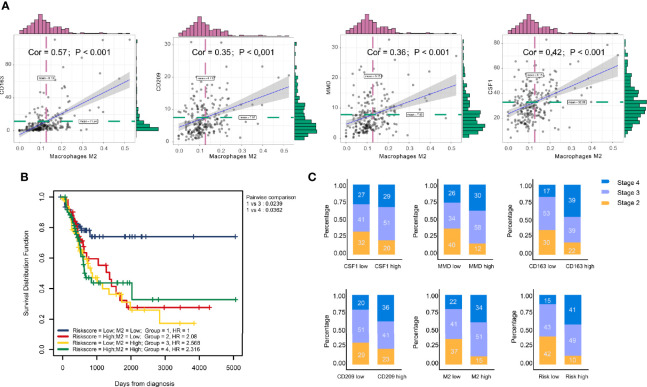
Clinical phenotype correlation. **(A)** Significant association between M2 macrophage frequency and co-expression genes are indicated. The correlation of CD163 was the highest at 0.57. **(B)** Combining infiltrating M2 macrophages elevated the predictive accuracy of risk score even more than either of them alone. The hazard of high ‘M2 + Risk score’ group showed more survival risk than the lower group (Kaplan–Meier analysis, M2 + risk score binary: HR = 2.316). **(C)** Clinical stage correlation to different expression level of CD163, CD209, CSF1, and MMD.

Significant associations between M2 macrophage frequency and co-expressed genes are indicated in **Figure 5A**; the correlation for CD163 was the highest at 0.57. Intriguingly, combining infiltrating M2 macrophages elevated the predictive accuracy of the risk score even more than either of them alone, the hazard of the high M2 + risk score group showed more survival risk than the lower group (Kaplan–Meier analysis, M2 + Risk score binary: HR = 2.316; **Figure 5B**). M2 macrophage proportions and risk were prognosis risk factors, and combination with our four-gene risk score for M2 macrophage proportions could elevate the predictive accuracy. Distribution of clinical stage parameters in co-expression genes low and high groups was performed in **Figure 5C**. In high expression groups for these four genes, the stages were higher, suggesting worse prognostic status in the high expression groups. In high expression groups of risk scores and M2 macrophages groups, the clinical stages were the similarly higher.

### TMB Correlation

**Figure 6:**
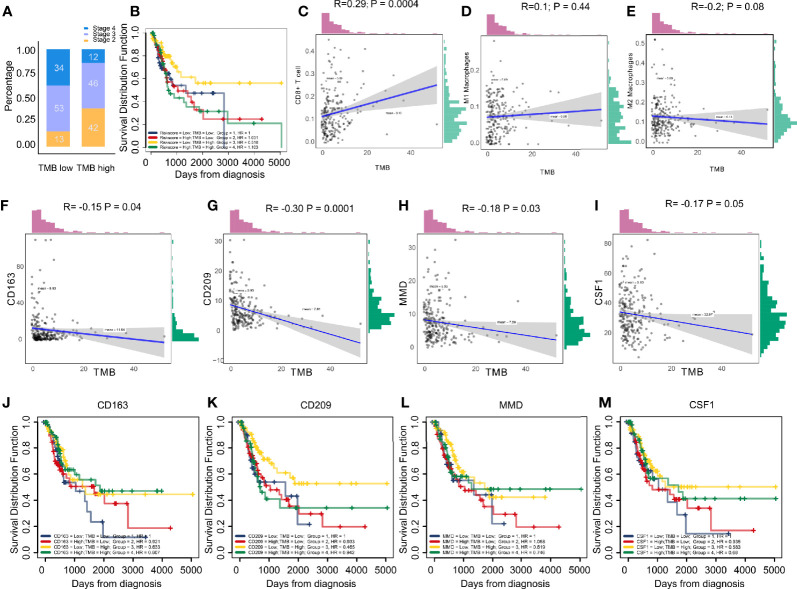
**(A)** Low tumor mutation burden acted as a protective factor in bladder cancer micro-environments. **(B)** Combining tumor mutation burden elevated the predictive accuracy of risk score even more than either of them alone. The hazard of “low TMB + high risk score” group showed more survival risk than the other group (Kaplan-Meier analysis, Low Risk score + High TMB; HR = 0.518). **(C–E)** The correlation between immune cells and tumor mutation burden. The results showed CD8+ T cells and type M1 macrophages positively correlated with tumor mutation burden, while the M2 macrophages were the opposite. **(F–I)** CD163, CD209, CSF1, and MMD negatively correlated with tumor mutation burden, clarifying the high level M2 macrophages in patients with low tumor mutation burden. **(J–M)** Combining tumor mutation burden elevated the predictive accuracy of CD163, CD209, CSF1, and MMD even more than either of them alone.

The stage in low TMB were more advanced, which means low levels of mutation acted as protective factors in BC micro-environment (**Figure 6A**). Combining TMB elevated the predictive accuracy of risk score even more than either of them alone. The hazard of the low TMB + high risk score group showed more survival risk than the other group (Kaplan–Meier analysis, low risk score + High TMB; HR = 0.518; **Figure 6B**). We, explored the correlation between immune cells and TMB and found that CD8+ T cells and M1 macrophages positively related to TMB, while M2 macrophages were the opposite (**Figures 6C–E**). CD163, CD209, CSF1, and MMD negatively related to TMB, which clarified the high levels of M2 macrophages in low-TMB patients (**Figures 6F–I**). Finally, combining TMB level elevated the predictive accuracy of CD163, CD209, CSF1, and MMD even more than either of them alone (**Figures 6J–M**).

### Pan-Cancer Analysis in TIMER

**Figure 7:**
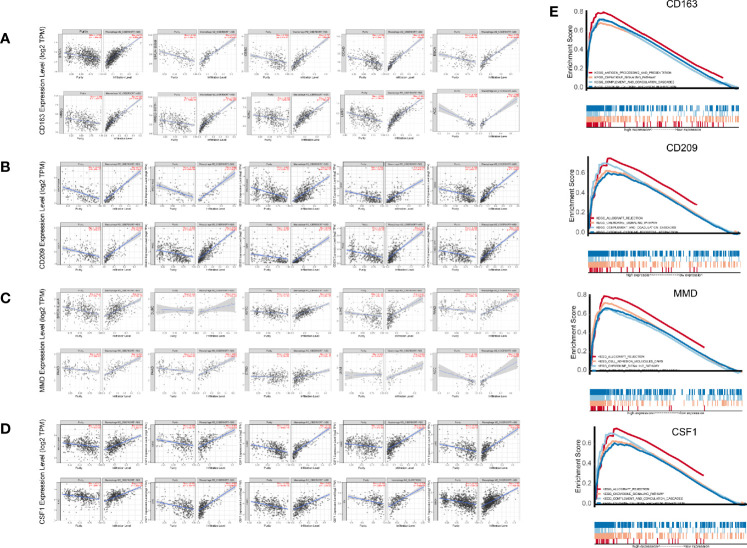
**(A–D)** Pan-cancer analysis in TIMER. **(E)** Gene set enrichment analysis (GSEA).

These results demonstrated the role of CD209, CD163, CSF1, and MMD in melanoma. Next, we analyzed the correlations between these co-expressed factors and M2 macrophage infiltration in other types of cancers. CD209, CD163, CSF1, and MMD were related to M2 macrophage proportions in cutaneous melanoma, breast carcinoma, head and neck squamous cell carcinoma, hepatocellular carcinoma, prostate cancer, renal cancer, and lung adenocarcinoma (**Figures 7A–D**).

### Gene Set Enrichment Analysis

GSEA analysis showed that antigen processing and presentation, the chemokine signaling pathway, complement and coagulation cascades, and cytokine-cytokine receptor interactions were related to the high expression group (**Figure 7E**). P-values were all less than 0.01. In addition, we found that these biological pathways were immune-related and were involved in tumor immunity.

## Discussion

**Figure 8:**
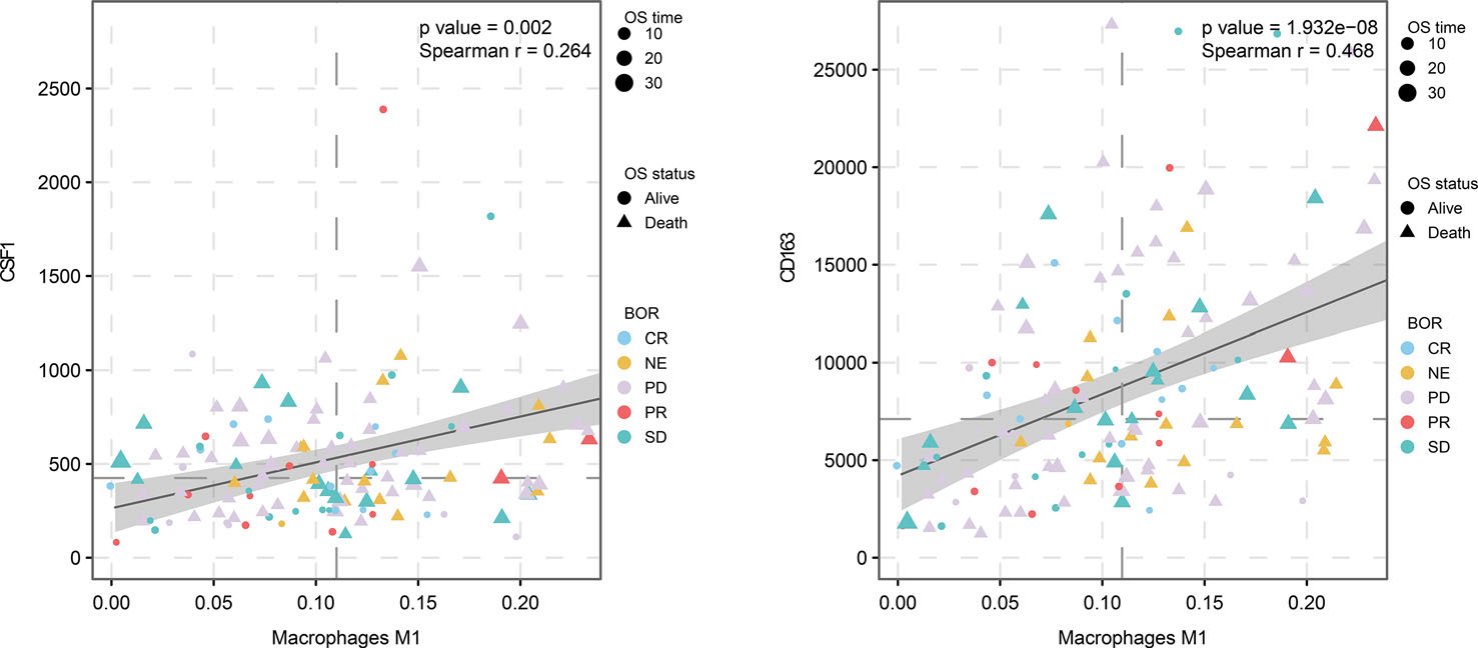
M2 macrophage proportion correlations for CD163 and CSF1 in the immunological therapy cohort.

In the tumor microenvironment, the mechanism of the functional difference between M2 macrophages and M1 macrophages is still not completely clear. The biological cytological role of M2/M1 macrophages in tumor tissues still needs to be explored. This article is based on the WGCNA algorithm and the CIBERSORT algorithm to mine the common part of the M2 macrophage co-expression network in two different queues. We also determined the association of these factors with macrophages in the cohort of immune checkpoint therapy (**Figure 8**). Then, by analyzing the correlation between these four genes and M0 and M1 macrophages, we found that these factors had the strongest correlation with M2, but had no significant relationship with other types of macrophages (**Supplementary Figure 1**). By analyzing this part of the intersection, we tried to explain the biological function of co-expressed genes with M2 macrophages and related pathway changes from the perspective of bioinformatics.

CD163, CD209, MMD, and CSF1 were identified as the genes most often co-expressed with M2 macrophages in TCGA-BLCA and GSE31189 cohorts. By GSEA analysis, antigen processing and presentation, the chemokine signaling pathway, complement and coagulation cascades, and cytokine-cytokine receptor interactions were related to the high expression group. M1 macrophages tend to a Th1 response gene expression pattern, and secrete nitric oxide, IL-1, IL-6, IL-12, CCL2, CCL3, CXCL9, CXCL10, and various cytokines. They also pass the rich expression of MHC II and B7 molecules to present antigen efficiently (24). This is a kind of protection against pathogen invasion, monitoring tumor pathological changes, and participation of type Th1 immune response of macrophages (25). However, M2 macrophages have poor tumor antigen processing ability.

The protein encoded by CD163 is a member of the SRCR superfamily, which is only expressed in monocytes and macrophages and has been widely used to label macrophages (26). Macrophages have plasticity, and investigators have developed biomarkers for identifying cell subtypes of macrophages (27). Scavenger receptors CD163 (hemoglobin–haptoglobin SCR), CD204 (scavenger receptor A), and CD206 (mannose receptor C type 1) are markers of M2-macrophages (28). CD163 showed a significant association with worse OS in cancer patients, except for lung and liver cancer patients. CD209 encodes c-type lectin, which plays a role in cell adhesion and pathogen recognition (29, 30). Recognition of this receptor has a major impact on public health. The protein is composed of four domains, including a c-terminal carbohydrate recognition domain, a flexible tandem repeat neck domain, a transmembrane region, and an n-terminal domain. The plasmin domain is involved in internalization (29, 30). This gene is expressed in M2 type macrophages and is widely used as a biomarker for M2 macrophages. Monocyte-to-macrophage differentiation (MMD) refers to attributes expressed in mature macrophages but not monocytes. Recent studies have shown that MMD mRNA can be detected in macrophages in almost all tissues (31). The activation of macrophages is regulated by environmental signals and endogenous programs. Liu et al. found that expression levels of MMD were significantly increased during monocyte differentiation (32). In addition to PAQR family proteins, MMDs promote macrophage activation (32). Colony stimulating factor 1 (CSF1) is a cytokine that regulates the proliferation, differentiation, and biological functions of macrophages. Lin et al. targeted TAMs by inhibiting bone marrow cell receptor colony stimulating factor-1 receptor (CSF1R) to reduce the number of tumors initiating cells and inhibiting metastasis (33). These results provide reliable evidence for our bioinformatic method. There were 16 other M2 macrophage-related genes in the intersecting areas that require deep exploration, including CD14 and VSIG4.

This study has some limitations. We only used intersection data of two queues and no cross-validation of multicenter data. In addition, there was a lack of experimental verification of M2 macrophage biomarkers in the intersection.

In summary, we identified CD163, CD209, CSF1, and MMD as biomarkers of M2 macrophages by constructing a proportional co-expression network of immune infiltrated cells, and proposed 16 candidate related factors. The related markers and biological processes of M2 macrophages in the immune micro-environment are revealed from the perspective of bioinformatics, which provides a new approach to explore the macrophage polarization.

## Data Availability Statement

Publicly available datasets were analyzed in this study. These data can be found here: The TCGA-BLCA dataset used in this study could be obtained from TCGA database (https://cancergenome.nih.gov/). GEO datasets (GSE31189) used in this study could be obtained from GEO database (https://www.ncbi.nlm.nih.gov/geo/). genomic, transcriptomic, and clinical information from patients with metastatic urothelial cancer treated with an anti-PD-L1 agent (atezolizumab) is obtained under the Creative Commons 3.0 license and can be downloaded from http://research-pub.gene.com/IMvigor210CoreBiologies.

## Ethics Statement

Ethical review and approval was not required for the study on human participants in accordance with the local legislation and institutional requirements. Written informed consent for participation was not required for this study in accordance with the national legislation and the institutional requirements.

## Author Contributions

YW, KY, and JB designed the study. JL and JW searched, collected and pre-processed data. YW and KY analyzed and wrote the manuscript. All authors contributed to the article and approved the submitted version.

## Funding

This work was supported by China Shenyang Science and Technology Plan (20–205–4–015).

## Conflict of Interest

The authors declare that the research was conducted in the absence of any commercial or financial relationships that could be construed as a potential conflict of interest.

## Supplementary Material

Supplementary Figure 1 Analysis of the correlation between four genes (CD209, CD163, MMD, and CSF1) and M0 and M1 macrophages. These factors had the strongest correlations with M2, but had no significant relationship with other types of macrophages.

## Abbreviations

TCGA, The Cancer Genome Atlas; GEO, Gene Expression Omnibus, BLCA, Bladder urothelial carcinoma; ROC, Receiver operating characteristic; AUC, Area under the curve; HR, Hazard ratio; TME, Tumor microenvironment.
