# Supplementary figures and images for: M2 Macrophage Co-Expression Factors Correlate With Immune Phenotype and Predict Prognosis of Bladder Cancer
Source: Front Oncol. 2021 Mar 22;11:609334. doi: 10.3389/fonc.2021.609334 (PMC8019942; doi:10.3389/fonc.2021.609334)

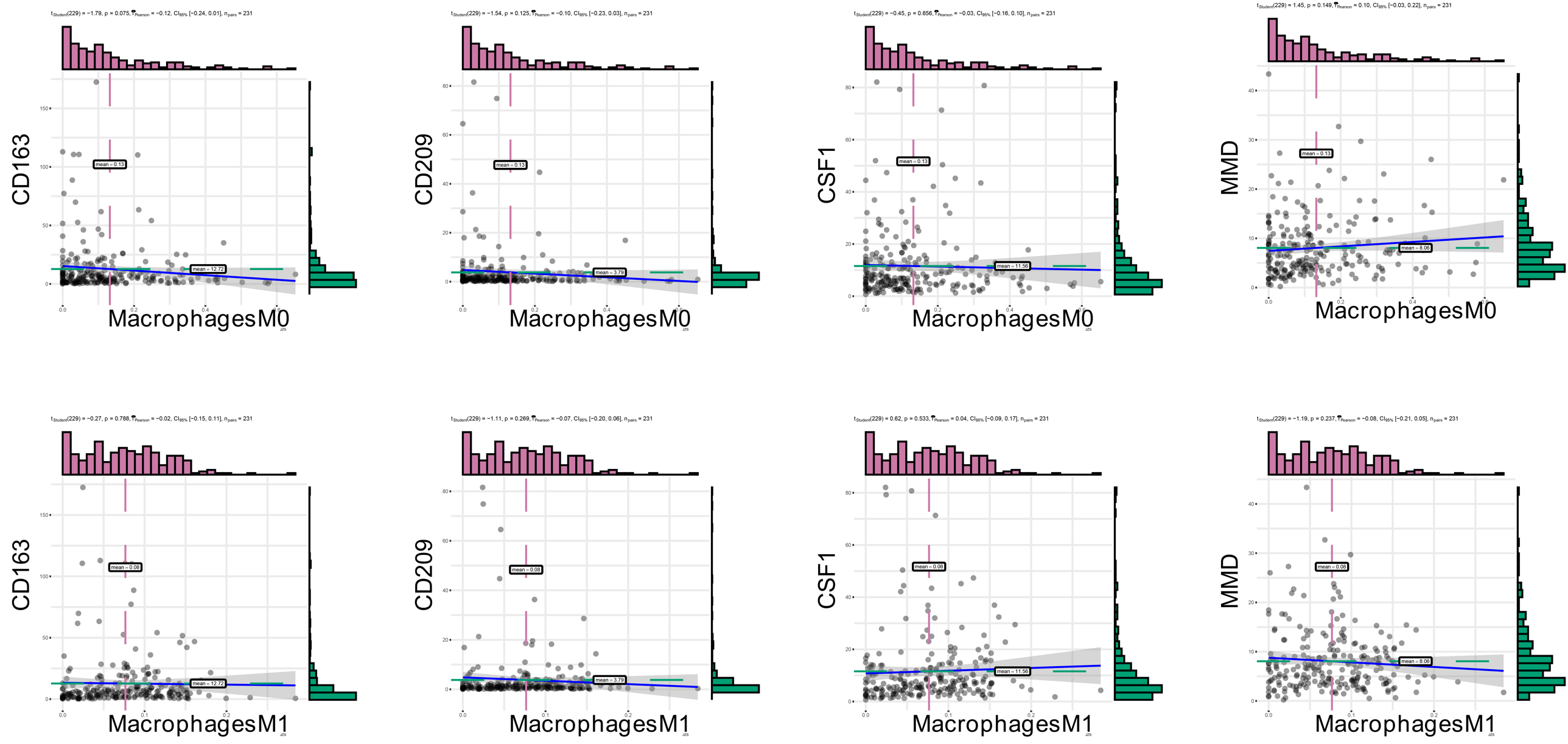

Supplement: Supplementary Figure 1 — Analysis of the correlation between four genes (CD209, CD163, MMD, and CSF1) and M0 and M1 macrophages. These factors had the strongest correlations with M2, but had no significant relationship with other types of macrophages. [file Image_1.tif]
